# Supplementary material for: Electric-field-induced non-ergodic relaxor to ferroelectric transition in BiFeO3–xSrTiO3 ceramics
Source: J Mater Chem C Mater. 2023 Apr 14;11(21):6902–11. doi: 10.1039/d2tc05100a (PMC10273322; doi:10.1039/d2tc05100a)
Supplement: TC-011-D2TC05100A-s001 [file TC-011-D2TC05100A-s001.pdf]

Electronic Supplementary Material (ESI) for Journal of Materials Chemistry C.
This journal is © The Royal Society of Chemistry 2023

Supporting information

Electric-field-induced non-ergodic relaxor to ferroelectric transition in $BiFeO_3$-$x$$SrTiO_3$ ceramics

Leonardo Oliveira,[1] Jeppe Ormstrup,[1] Marta Majkut,[2] Maja Makarovic,[3,4] Tadej Rojac,[3,4] Julian Walker,[5] Hugh Simons[1, *]

[1]*Department of Physics, Technical University of Denmark, 2800 Kgs. Lyngby, Denmark*
[2]*ESRF – The European Synchrotron, Avenue des Martyrs, 38000 Grenoble, France*
[3]*Electronic Ceramics Department, Jožef Stefan Institute, 1000 Ljubljana, Slovenia*
[4]*Jožef Stefan International Postgraduate School, 1000 Ljubljana, Slovenia*
[5]*Department of Materials Science and Engineering, Norwegian University of Science and Technology, Trondheim, Norway*

Corresponding Author: leosdo@dtu.dk, husimo@fysik.dtu.dk

Content

$\varepsilon(E,t)$ dependency S2

Dielectric permittivity S3

$\varepsilon(E,t)$ dependency

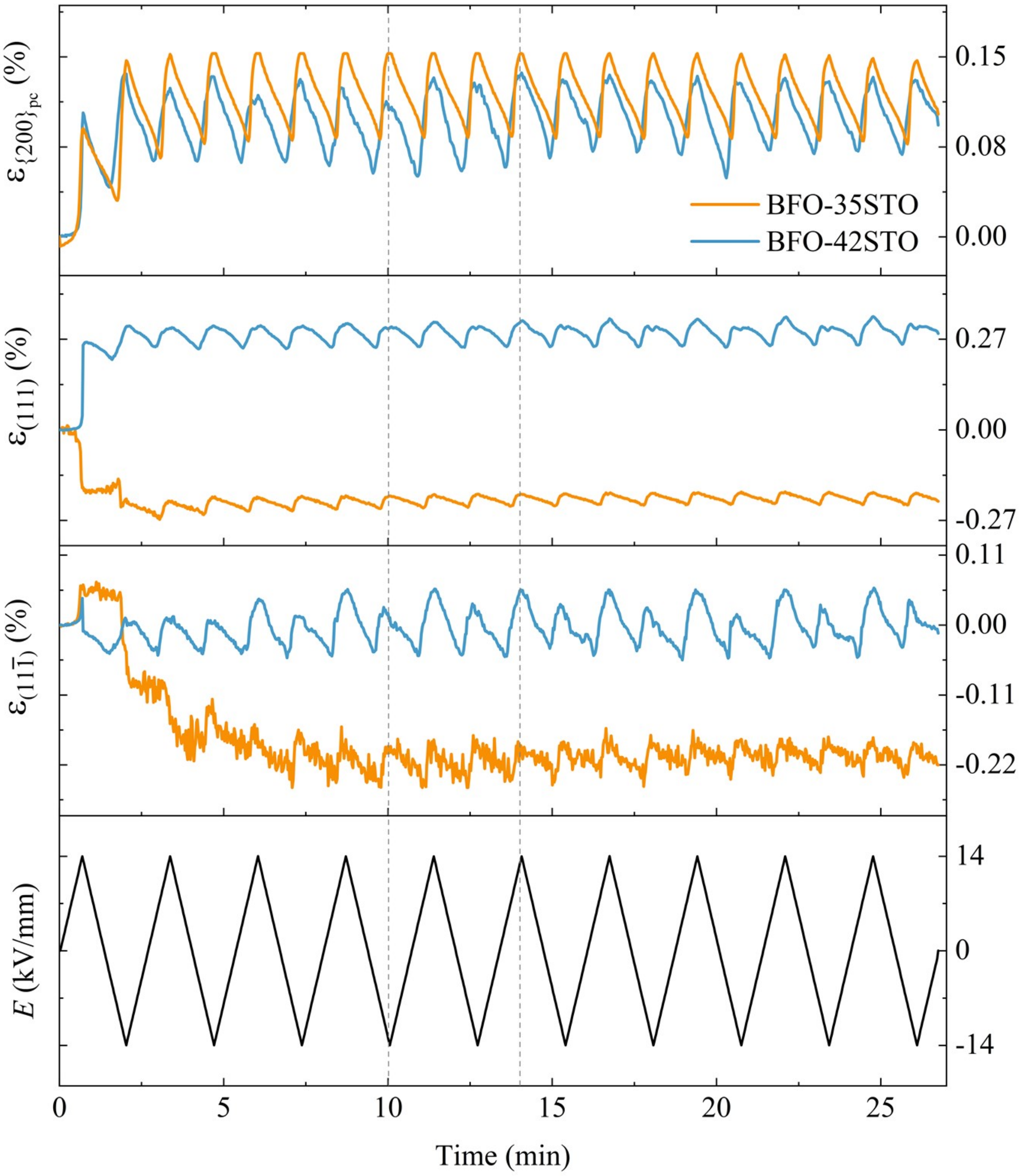

Figure S1. Comprehensive time and E-field evolution of $\varepsilon_{\{111\}_{pc}}$ and $\varepsilon_{\{200\}_{pc}}$ for the entire field cycling. The $\varepsilon(E,t)$ dependency shows a general behavior for times higher than the initial wake-up cycle. This trend is exemplified from the dashed lines at 10 min and 14 min. One may conclude that $\varepsilon_{\{200\}_{pc}} > \varepsilon_{(11\bar{1})} \approx \varepsilon_{(111)}$ for BFO-35STO, while $\varepsilon_{(111)} > \varepsilon_{\{200\}_{pc}} > \varepsilon_{(11\bar{1})}$ for BFO-42STO.

Dielectric permittivity

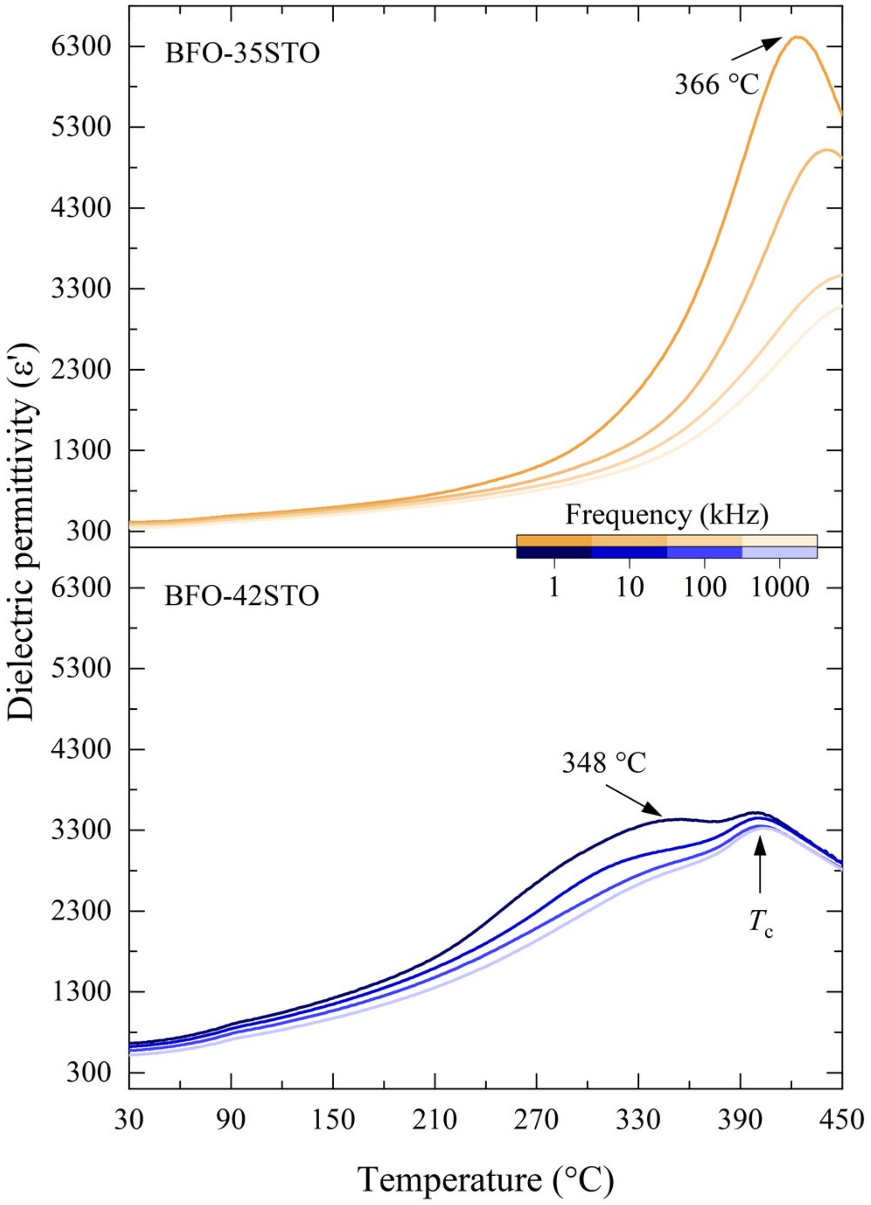

Figure S2. Temperature dependent dielectric permittivity for BFO-35STO and BFO-42STO at selected frequencies. The frequency dependent peak is characteristic of the relaxor behavior. The arrows at 1Hz illustrate the peak shift towards lower temperatures, indicative of the increased ergodicity in BFO-42STO sample. Increasing the STO fraction the Curie temperature (T_C) is shifted to 401 °C, as seen by the frequency independent peak in BFO-42STO.
